# Multivariate analysis of clinicopathological and prognostic significance of miRNA 106b~25 cluster in gastric cancer

**DOI:** 10.1186/s12935-019-0918-7

**Published:** 2019-07-29

**Authors:** Fangxuan Li, Jinchao Huang, Juntian Liu, Wengui Xu, Zhiyong Yuan

**Affiliations:** 1 Department of Radiotherapy, Tianjin Medical University Cancer Institute and Hospital, National Clinical Research Center for Cancer, Tianjin’s Clinical Research Center for Cancer, Key Laboratory of Cancer Prevention and Therapy, Huanhuxi Road, Hexi District, Tianjin, 300060 China; 2 Department of Cancer Prevention, Tianjin Medical University Cancer Institute and Hospital, Huanhuxi Road, Hexi District, Tianjin, 300060 China; 3 Department of Molecular Imaging and Nuclear Medicine, Tianjin Medical University Cancer Institute and Hospital, Huanhuxi Road, Hexi District, Tianjin, 300060 China

**Keywords:** miRNA 106b, miRNA 93, miRNA 25, Gastric cancer, PCA, PLS-DA

## Abstract

**Background:**

miRNA 106b~25 cluster were demonstrated to be an oncogene. In previous study, we had analyzed the diagnostic significance of miRNA 106b~25 based on its carcinogenesis effect. The significance of miRNA 106b~25 for prognosis of gastric cancer were not researched.

**Methods:**

We applied multivariate analysis of PCA, PLS-DA and Cox Regression for clinicopathological features and survival time to explore the significance of miRNA 106b~25 expression in plasma and cancer tissues for gastric cancer.

**Results:**

The expression of miRNA 106b, miRNA 93 and miRNA 25 in plasma were positively correlated with their expression in tumor tissues. Via PCA analysis, it was found that miRNA 106b~25 expression in plasma and tumor, T, N and TNM stage were correlated with each other. Via PLS-DA analysis, we identified that T, N and TNM stage were important factors for miRNA 106b~25 expression both in plasma and tumor (all VIP value > 1.2). According to loading weights of variables for the first and second components, it was found that the importance of the miRNA 106b~25s expression carried with the progressed stage of gastric cancer. In the survival analysis, COX regression showed that T stage, plasma miRNA 106b and tumor miRNA 93 were significant risk factors for overall survival [HR: 0.400 (0.205–0.780); *P *= 0.007; HR: 0.371 (0.142–0.969), *P *= 0.043; 0.295 (0.134–0.650), *P *= 0.002].

**Conclusion:**

Plasma and tumor miRNA 106b~25 expression correlated with T, N and TNM stage. Increased miRNA 106b~25 expression was important characters carried with gastric cancer progression. T stage, plasma miRNA106b and tumor miRNA 93 significant risk factors for overall survival.

**Electronic supplementary material:**

The online version of this article (10.1186/s12935-019-0918-7) contains supplementary material, which is available to authorized users.

## Background

miRNA 106b~25 cluster were demonstrated to be an oncogene, which promote malignant cell proliferation, migration, invasion as well as tumor angiogenesis [1, 15]. The miRNA 106b~25 polycistronic contain three pre-miRNAs including highly conserved miRNA 106b, miRNA 93 and miRNA 25 [9]. These three miRNAs are targeted in the intron 13 of 515-bp region of chromosome 7q22 for gene MCM7 and present active transcription in the MCM7 primary RNA transcription [6, 15]. This cluster has been reported to be over-expressed in many cancers, such as esophageal cancer [21], prostate cancer [7, 16], non-small cell lung cancer [17], and hepatic cell cancer [20]. The trigger targets of their oncogenic process involving E2F1 and TGF-β, as well as Retinoblastoma protein (RB) gene, tumor protein 53 (TP53) [23] and phosphatase and tension homolog deleted on chromosome (PTEN) have been demonstrated in regulating mechanism of miRNA 106b~25 [24].

In previous study, the miRNA 106b~25 cluster implement proliferative, anti-apoptotic, cell cycle-promoting proficiency in cell experiments and tumorigenicity in vivo [4, 22]. Petrocca’s study suggests that cancer cells can alternate action mechanisms of miRNA 106b~25 to facilitating cells regeneration and relieving apoptosis [14]. miRNA 106b~25 over-expression increased apoptosis through regulation of the tumor suppressor genes [8]. Furthermore, suppression of miRNA 25, 93 and 106b results in facilitation of G1/S phase transition and decreasing cell cycle G0/G1 phase arrest [8]. Many studies demonstrated that the miRNA 93 also play an important role in enabling angiogenesis in caner [3].

Gastric cancer is the fourth most common cancer and the second leading cause of cancer related death in the world wide [18]. It had demonstrated that miRNA 106b and miRNA 93 were upregulated in primary tumors and highly expressed in all gastric cancer cell lines [5, 10]. miRNA 106b~25 clusters may take part in tumorigenesis, progression of gastric cancer via negative regulate E2F1, TGF-β pathway [4, 9, 10, 14]. Tumor suppressing gene RB and PTEN and oncogene P21 and Bim [8] also were the direct targets of miRNA 106b~25. So, many studies suggested that the miRNA 106b~25 may be an intrinsic factor of gastric carcinogenesis. In large-scale analysis, the plasma concentrations of miRNA 106b were significantly higher in gastric cancer patients, and significantly decreased in pre-operative serum compared with post-operative serum [13]. In TCGA database, all these three miRNAs were significantly higher expressed in cancer when comparing with normal tissues (Additional file 1: Table S1).

In our vitro study, inhibiting miRNA 106b∼25 cluster via transfecting antisense RNA can influence proliferation, migration, and invasion, G0G1 phase arrest of gastric cancer cells [26]. In clinic, we found that three components of miRNA 106b~25 cluster expressed consistently at a high level both in tumor specimens and plasma, and associated with clinical pathological factors [27]. When comparing the diagnostic efficacy, plasma miRNA 106b was significantly higher diagnostic efficacy than CA724, CA242 and CA199 [2], the diagnostic efficacies of miRNA 93 and miRNA 25 were significantly higher than CA199 [11]. However, the significance of miRNA 106b~25 for prognosis of gastric cancer were not researched. As well as in TCGA data base (Additional file 1: Table S1), the survival significance was not observed due to many studies lacking survival data. In this study, we examined 60 gastric patients’ miRNA 106b~25 expression level in plasma and cancer tissues. Then analyzed the association between miRNA 106b~25 expression and clinicopathological features and overall survival of gastric cancer patients after 5-years following-up.

## Methods

### Patients

We collected 60 gastric cancer tissues samples from March to May in 2013 at Tianjin Medical University Cancer Institute and Hospital, each one paired with non-tumor gastric tissue(at least 5 cm away from the edge of tumor, and there was no tumor cell confirmed by a pathologist [25, 30]) and perioperative peripheral blood sample from the same patient. Inclusive criteria were: (1) gastric cancer patients received radical resection for stomach tumor. (2) all histological diagnosis was confirmed as gastric adenocarcinoma. All clinical data were analyzed according to the 8th stomach cancer tumor-node-metastasis (TNM) staging classification of the Union of International Control Cancer (UICC) [28]. (3) all patients had not received chemotherapy or radiotherapy before collecting samples. (4) all patients had not chronic disease or infectious diseases or history of other malignancy.

Informed consent was taken from every subject, and the Human Research Ethics Committee of Tianjin Medical University approved all aspects of this study.

### Samples

All tissues samples were preserved in liquid nitrogen after removal from human body. Blood samples were obtained immediately following diagnosis and prior to any oncological treatment. The peripheral blood (5 mL) samples were collected into ethylenediaminetetraacetic acid (EDTA) anticoagulative tubes immediately. After collection, the blood samples were subjected for isolation of cell-free nucleic acids by using a three-spin protocol (2000*g* for 30 min, 4000*g* for 5 min, 8000*g* for 5 min) to prevent contamination from cellular nucleic acids.

### RNA extraction and detection of miRNAs

Total RNA of tissues was extracted by using Trizol (Invitrogen, USA); Plasma RNA was extracted by using acid phenol according to the manufacturer’s instructions. Total RNA was quantified by microfluidics analysis (Gene Quant, Switzerland). The amounts of miRNAs were quantified in duplicate by quantitative reverse transcription polymerase chain reaction (RT-PCR) using the human TaqMan MicroRNA Assay Kits (Applied Biosystems, Foster City, CA, USA). After the reverse transcription reaction which was carried out with TaqMan MicroRNA Reverse Transcription Kit (Applied Biosystems), cDNA solution was amplified using TaqMan Universal PCR Master MixII with no Amp Erase UNG (Applied Biosystems). RT-PCR was run on 7500 Real Time PCR system (Applied Biosystems), and the cycle threshold (Ct) values were calculated with the SDS 1.4 software (Applied Biosystems). All reactions were performed in triplicate.

Through the 2^−ΔΔCt^ method, expressions of miRNAs by U6, while the expressions of miRNAs from tissues samples were normalized by miRNA 39 according to the manufacturer’s instructions. The Ct was calculated by subtracting the Ct values of reference substance from the Ct values of the interesting miRNAs. Mean Ct and standard deviation values were calculated without outliers (i.e., replicates with Ct differing by more than one cycle from the median). The ΔΔCt was then calculated by subtracting ΔCt of the median of control samples from ΔCt of study group. Fold change was calculated by the equation 2^−ΔΔCt^ [24]. Then, we used division calculation to achieve the 2^−ΔΔCt^ multiple between gastric cancer tissues and one to one correspondence adjacent non-tumorous tissues.

### Statistical methods

Statistical analyses were performed using the SPSS software package (version 16.0; SPSS, Chicago, IL, USA). Two-tailed *P*-values of less than 0.05 were considered to statistically significant difference. Continuous variable was described by median and mean ± standard deviation ($\bar{x} \pm {\text{s}}$). Independent sample *t* test was used in comparison of continuous variables. The Chi squared and Fisher exact tests were applied in categorical variable for univariate analysis.

All clinical features were set as variable X to establish the matrix. Then SIMCA-P 13.0 (Umea, Sweden) was applied in matrix analysis. Principal component analysis (PCA) was used for profile analysis; meanwhile, partial least-squares discriminant analysis (PLS-DA) was used to confirm the significant variable for the categories. Loading plot and Variable Importance for the Projection (VIP) was used to determine the significant important variable for the miRNA 106b~25 (VIP > 1.2) [7].

## Results

### Expression of miRNA106b~25 in plasma and cancer tissues in gastric cancer

**Fig. 1:**
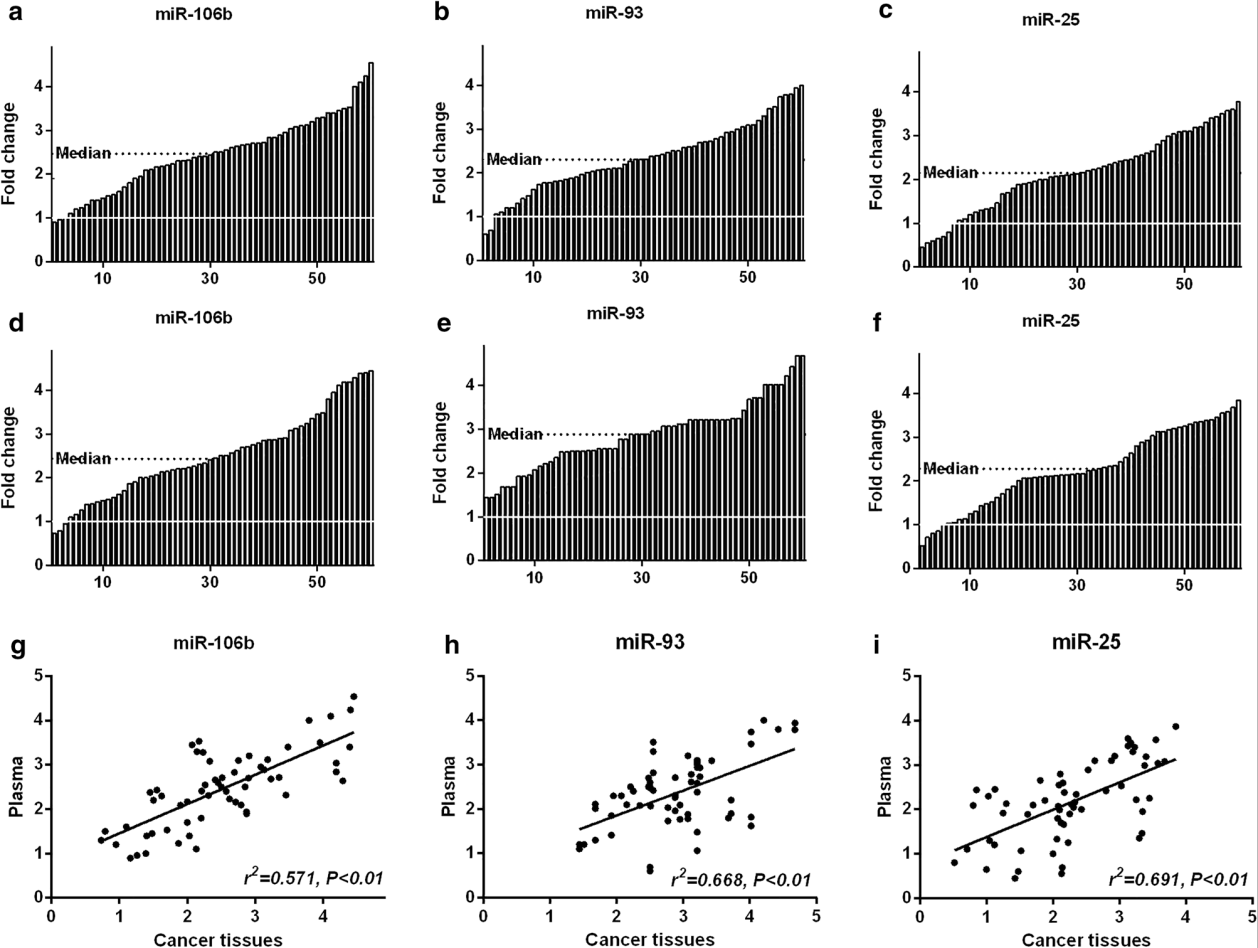
The expression of plasma and tumor miRNA 106b~25 in gastric cancer. **a**–**c** showed sequence diagram of the fold change of plasma miRNA 106b, miRNA 93 and miRNA 25 expression. **d**, **e** Sequence diagram of the fold change of miRNA 106b, miRNA 93 and miRNA 25 expression in tumor tissues. **g**–**i** Correlation analysis of plasma and tumor tissues miRNA106b~25

Figure 1a–c showed sequence diagram of the fold change of plasma miRNA 106b, miRNA 93 and miRNA 25, which set the fold change of “1” as cutting line. The median fold change of plasma miRNA 106b, miRNA 93 and miRNA 25 were 2.465, 2.305 and 2.145, respectively, the mean fold change of plasma miRNA 106b, miRNA 93 and miRNA 25 were 2.457 ± 0.856, 2.3512 ± 0.796, 2.162 ± 0.846 (Table 1).

**Table 1:** The summary of clinicopathological features and miRNA106b~25 of gastric cancer patients

Clinicopathological features	
Age (mean ± SD, year)	53.75 ± 9.76
Sex (male/female)	53/7
Location (proximal/middle/distal)	16/12/32
Tumor size (< 5 cm, ≥ 5 cm)	26/34
Bormann type (I/II/III/IV)	5/14/31/10
T stage (T1/T2/T3/T4)	3/13/4/40
N stage (N0/N1/N2/N3)	19/18/9/14
TNM stage (I/II/III/IV)	12/12/31/5
Histological grade (well/poor differentiated)	17/43
Plasma miRNA 106b (median, range)	2.465 (0.90–4.54)
Plasma miRNA 93 (median, range)	2.305 (0.60–4.00)
Plasma miRNA 25 (median, range)	2.145 (0.45–3.87)
Tumor miRNA 106b (median, range)	2.430 (0.73–4.45)
Tumor miRNA 93 (median, range)	2.880 (1.44–4.68)
Tumor miRNA 25 (median, range)	2.165 (0.52–3.85)

Then, we determined the levels of miRNA 106b, miRNA 25 and miRNA 93 relative to the paired normal tissues. Figure 1d–f showed sequence diagram of the fold change relative to adjacent non-tumorous tissues. The median fold change of tumor miRNA 106b, miRNA 93 and miRNA 25 were 2.430, 2.880, 2.165, respectively, which was significantly increased expression. The mean fold change of tumor miRNA 106b, miRNA 93 and miRNA 25 were 2.516 ± 0.971, 2.885 ± 0.785, 2.275 ± 0.867.

Figure 1g–i showed the correlation analysis of plasma and tumor miRNA106b~25, the plasma miRNA 106b, miRNA 93 and miRNA 25 were positive correlated to tumor expression, respectively (*r*^2^= 0.571, *P *< 0.01; *r*^2^= 0.668, *P *< 0.01; *r*^*2*^= 0.691, *P *< 0.01).

### The multivariate analysis of the correlation between miRNA106b~25 and clinicopathological features

**Fig. 2:**
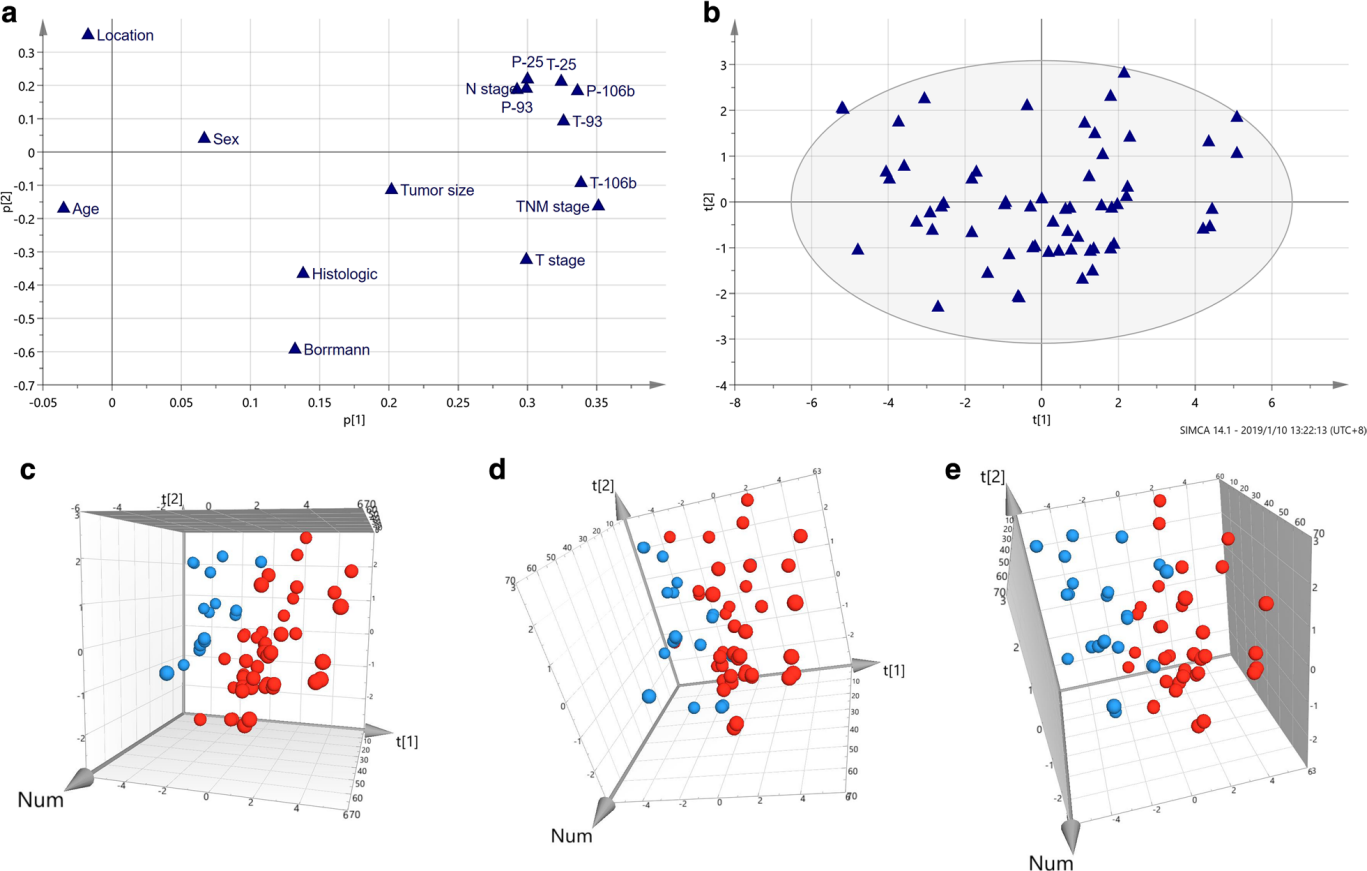
PCA analysis for clinicopathological features and miRNA 106b~25 in gastric cancer. **a**, **b** Loading plot and score scatter in PCA analysis. **c**–**e** 3D score plot according to T stage, N stage, TNM stage in PCA, Blue: T1 + T2, N0, TNM I + II stage, respectively; Red: T3 + T4, N1–N3, III + IV stage, respectively

Clinicopathological features and miRNA 106b~25 expressions were set as variable X to establish the matrix for PCA analysis. As shown in Fig. 2a, b of loading plot and score scatter, Principal component 1 (PC1) and PC2 explained 54.1% and 44.2% variation, the total of 99.3% variation were explained by these two-PCs. Loading plot shows how the X-variables vary in relation to each other, variables near each other are positively correlated, variables symmetric about the origin to each other are negatively correlated, the variables situated at 90 degrees from each other are almost uncorrelated in these 2 components. Thus, the plasma and tumor miRNA 106b~25 expression, T stage, N stage, TNM stage were correlated each other.

**Fig. 3:**
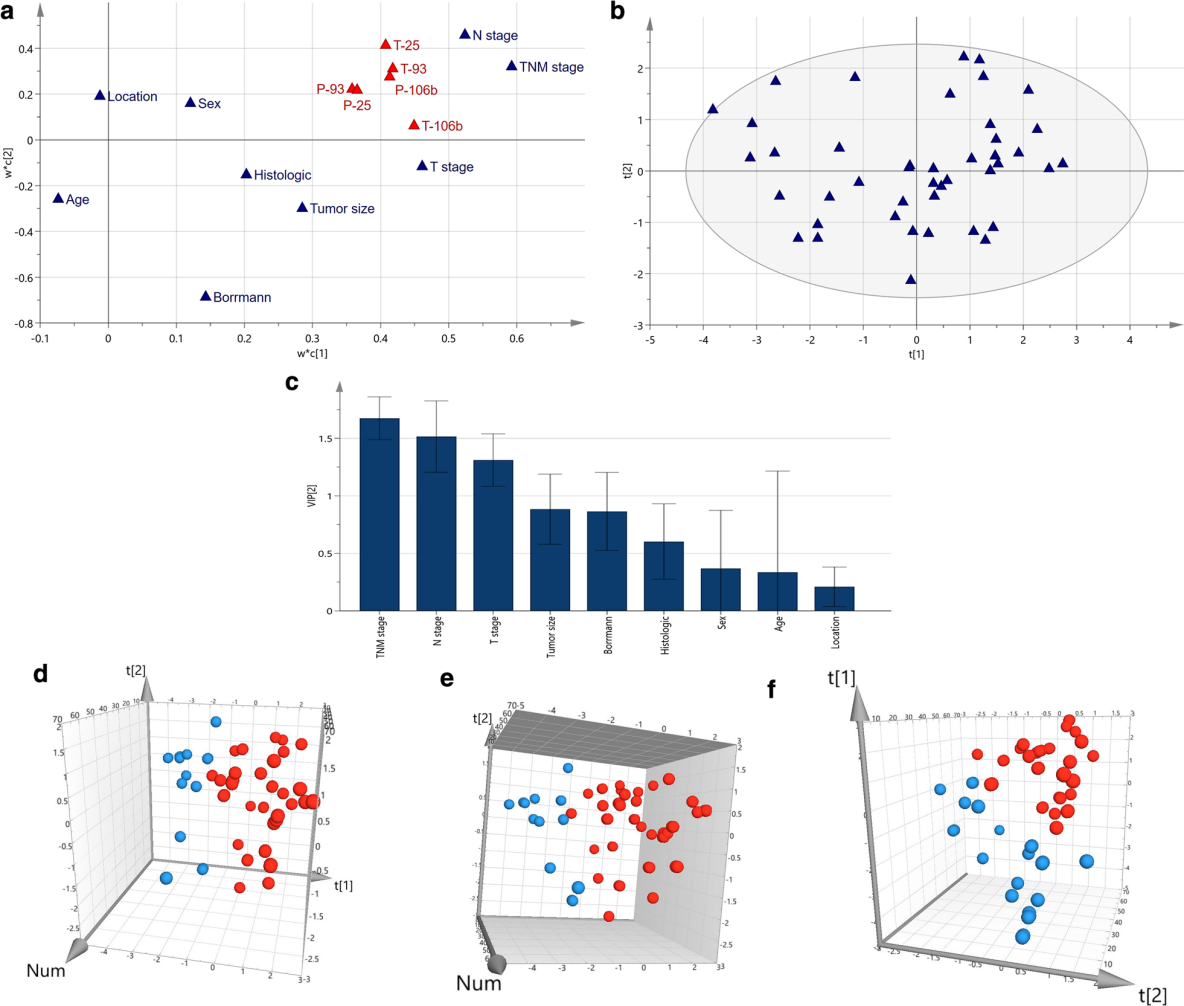
PLS-DA analysis for clinicopathological features and miRNA 106b~25 in gastric cancer. **a**, **b** loading plot and score scatter in PLS-DA analysis. **c** VIP value of clinicopathological features for miRNA 106b~25 expression. **d**–**f** 3D score plot according to T stage, N stage, TNM stage in PLS-DA, Blue: T1 + T2, N0, TNM I + II stage, respectively; Red: T3 + T4, N1–N3, III + IV stage, respectively

Clinicopathological features were set as variable X to establish the matrix for PLS-DA analysis. The explain rate of first two PCs, t1 and t2 was 43.4% and 54.4%, respectively. In PLS-DA analysis, X-variables with large w*’s (positive or negative) are highly correlated with miRNA 106b~25 expression (Y). These variables with large w*’s are situated far away from the origin (on the positive or negative side) on the plot (Fig. 3a, b). Thus, T stage, N stage, TNM stage were correlated with plasma and tumor miRNA 106b~25 expression. We also identified that T stage, N stage, TNM stage were important factors to plasma and tumor miRNA 106b~25 expression via VIP(VIP value = 1.67482, VIP value = 1.31006, VIP value = 1.51526, Fig. 3c).

Both PCA and PLS-DA score plot (Figs. 2c–e, 3d–f) showed that all point can be disguised by T stage, N stage, TNM stage. T3–T4 stage, N1–N3 stage, III-IV stage distributed on the right, while T1–T2 stage, N0 stage, I–II stage distributed on the left according to their first and second PCs loading weight. However, such PCs are mainly correlated to the TNM stage, tumor and plasma miRNA 106b, tumor miRNA 93, tumor miRNA 25 (loading weights > 0.3, first component), T stage and plasma miRNA25 (loading weights > 0.3, second component) in PCA analysis. In PLS-DA analysis, first component is mainly correlated to the N stage, TNM stage, T stage, tumor miRNA106b, miRNA 25 and miRNA93 (loading weights > 0.4), second component are mainly correlated to N stage, tumor miRNA25, tumor miRNA93 (loading weights > 0.3, shown in Table 2). This result confirms the importance of the miRNAs 106b~25 expression carried with the progressed stage of gastric cancer.

**Table 2:** Loading weights of all variables for the first and second components in PCA and PLS-DA analysis

Variables	PCA		PLS-DA	
	M1.p (1)	M1.p (2)	M1.w*c (1)	M1.w*c (2)
Plasma miRNA 106b	0.336298	0.181996	0.413691	0.274102
Plasma miRNA 93	0.292204	0.186027	0.358499	0.222264
Plasma miRNA 25	0.300041	0.316393	0.364789	0.215167
Tumor miRNA 106b	0.338569	− 0.0933063	0.44881	0.0596236
Tumor miRNA 93	0.326162	0.0910954	0.417487	0.308985
Tumor miRNA 25	0.324518	0.211675	0.407781	0.412313
Age	− 0.0350196	− 0.170789	− 0.073876	− 0.260368
Sex	0.0664001	0.040216	0.120416	0.160827
Location	− 0.0172643	0.252373	− 0.0127198	0.189534
Bormann type	0.132391	− 0.593899	0.142949	− 0.68586
Tumor size	0.201582	− 0.114009	0.284582	− 0.299471
Histological type	0.137613	− 0.367822	0.202056	− 0.151812
T stage	0.2992	− 0.32468	0.460746	− 0.116775
N stage	0.29889	0.189293	0.523468	0.458886
TNM stage	0.351118	− 0.162562	0.592729	0.320023

### Prognostic analysis

**Fig. 4:**
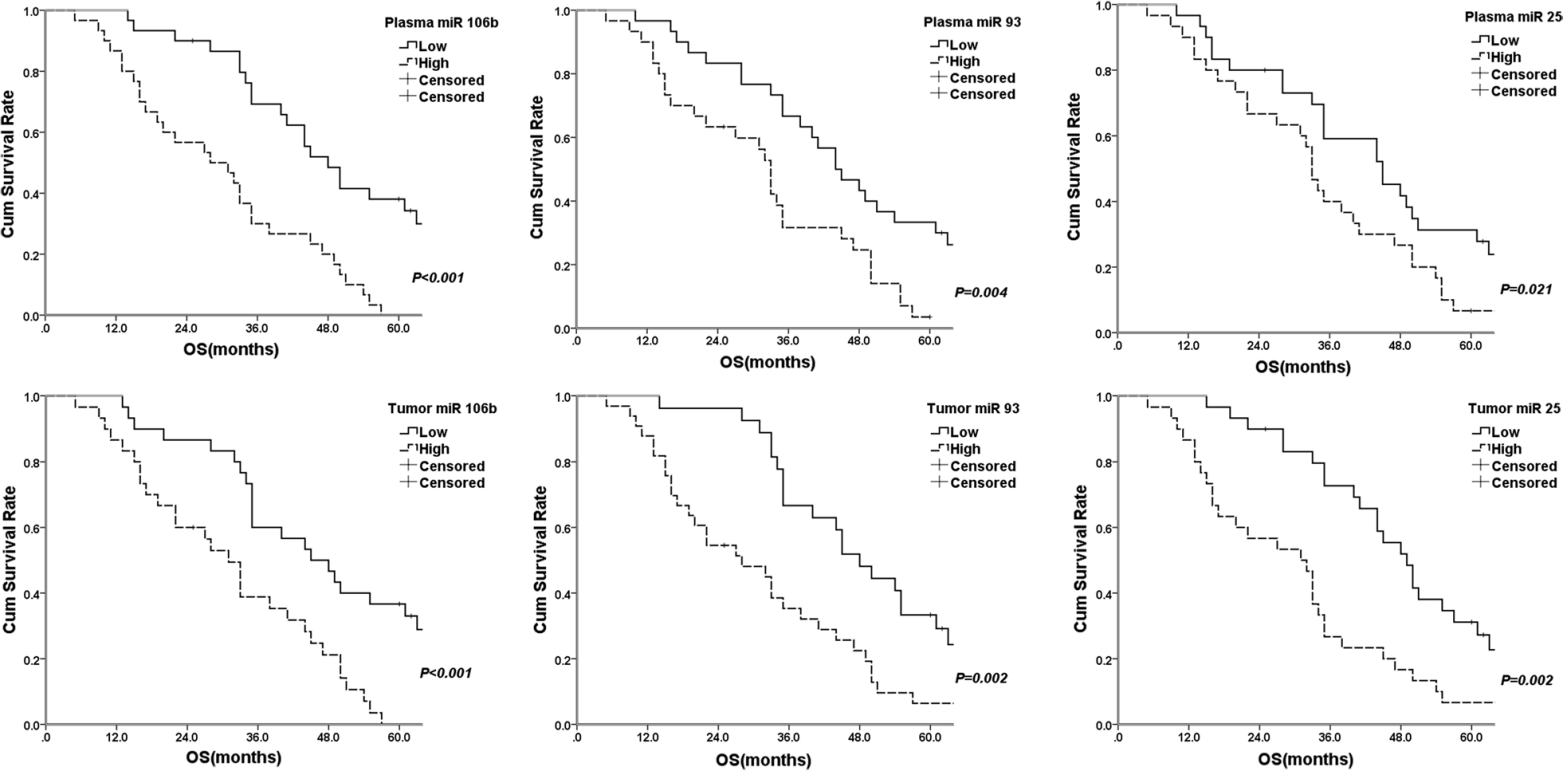
The survival curves according to plasma and tumor miRNA106b~25 of gastric cancer patients

In the survival analysis, tumor size (*P *< 0.01), T stage (*P *= 0.015), N stage (*P *< 0.01), TNM stage (*P *< 0.01), plasma miRNA 106b (*P *< 0.001), plasma miRNA 93 (*P *= 0.004), plasma miRNA25 (*P *= 0.029), tumor miRNA 106b (*P *< 0.001), tumor miRNA 93 (*P *= 0.002), tumor miRNA25 (*P *= 0.002), was significantly correlated with OS (Fig. 4). The survival rates based on clinicopathological features and miRNA 106b~25 were list in Table 3. In Table 4, we used COX regression to get adjusted hazard ratios for prognosis. T stage, plasma miRNA 106b and tumor miRNA 93 are significant risk factors for overall survival [HR: 0.400 (0.205–0.780); *P *= 0.007; HR: 0.371 (0.142–0.969), *P *= 0.043; 0.295 (0.134–0.650), *P *= 0.002].

**Table 3:** 1,3 and 5 year-survival rate of gastric cancer patients according to clinicopathological features and miRNA 106b~25

Clinicopathological features	Total	OS				
		1-YSR	3-YSR	5-YSR	x^2^	P
Age						
< 55	20	95.0	42.9	0.00	2.951	0.086
≥ 55	40	92.5	52.5	27.5		
Sex						
Male	53	94.3	50.3	21.3	1.945	0.163
Female	7	85.7	42.9	0.00		
Tumor location						
Proximal	16	93.8	56.3	18.8	3.271	0.105
Middle	12	91.7	33.3	0.00		
Distal	32	90.6	52.3	7.9		
Tumor size (cm)						
< 5	26	96.2	73.1	38.5	18.001	0.000
≥5	34	91.2	31.0	3.1		
Bormann type						
I + II	19	95.2	52.4	23.8	0.772	0.380
III + IV	41	92.3	47.9	0.160		
T stage						
T1, T2	16	93.8	75.0	43.8	5.914	0.015
T3, T4	44	90.9	40.1	9.4		
N stage						
N0	19	94.7	89.5	47.4	19.460	0.000
N1–N3	41	90.2	30.5	5.1		
TNM stage						
I, II	24	95.8	83.3	41.7	24.450	0.000
III, IV	36	88.9	32.2	2.9		
Histological grade (-differentiated)						
Well	17	94.1	52.9	23.5	0.445	0.505
Poor	43	90.7	48.1	16.8		
Plasma miRNA 106b^a^						
Low	30	96.7	69.2	38.1	17.219	0.000
High	30	86.7	30.0	0.00		
Plasma miRNA 93^a^						
Low	30	96.7	66.7	33.3	8.401	0.004
High	30	90.0	31.7	3.5		
Plasma miRNA 25^a^						
Low	30	96.7	59.1	31.3	5.358	0.029
High	30	90.0	40.0	6.7		
Tumor miRNA 106b^a^						
Low	30	96.3	66.7	33.3	12.533	0.000
High	30	87.9	35.3	6.4		
Tumor miRNA 93^a^						
Low	30	96.3	66.7	33.7	9.318	0.002
High	30	87.9	35.3	6.4		
Tumor miRNA 25^a^						
Low	30	96.7	72.7	31.2	10.047	0.002
High	30	86.7	26.7	6.7		

*YSR* year survival rate

^a^Median was set as cut-off value; low was ≤ median; high was > median

**Table 4:** Multivariate COX regression for prognostic features of gastric cancer patients

Features	β	SE	Wald	df	P	HR (95% CI)
T stage						
T1, T2	− 0.916	0.341	7.229	1	0.007	0.400 (0.205–0.780)
T3, T4						
N stage						
N0	0.612	0.714	0.734	1	0.392	1.844 (0.455–7.470)
N1–N3						
TNM stage						
I, II	0.703	0.446	2.485	1	0.115	2.019 (0.843–4.839)
III, IV						
Tumor size						
< 5	0.832	0.494	2.839	1	0.092	2.297 (0.873–6.042)
> 5						
Plasma miR 106b						
Low	− 0.990	0.489	4.099	1	0.043	0.371 (0.142–0.969)
High						
Plasma miR 93						
Low	− 0.125	0.403	0.096	1	0.757	0.882 (0.400–1.946)
High						
Plasma miR 25						
Low	0.555	0.407	1.861	1	0.172	1.742 (0.785–3.866)
High						
Tumor miR 106b						
Low	0.220	0.377	0.340	1	0.560	1.246 (0.595–2.612)
High						
Tumor miR 93						
Low	− 1.220	0.403	9.162	1	0.002	0.295 (0.134–0.650)
High						
Tumor miR 25						
Low	− 0.029	0.477	0.004	1	0.951	0.971 (0.381–2.473)
High						

To get adjusted hazard ratios, COX regression was applied for adjusting significant covariate in Kaplan–Meier prognostic analysis

*β* regression coefficient, *SE* standard error, *Wald* wald Chi square; *df* degree of freedom, *HR* hazard ratio

## Discussion

Increasing studies has showed that miRNA 106b~25 cluster plays oncogenic roles in malignant disease. miRNA 106b~25 has been reported to be up-regulated in several cancers, including esophageal squamous cell carcinoma [21], breast cancer [19], hepatocellular carcinoma [20] et al. The regulation targets of miRNA 106b~25 involving E2F1 and TGF-β, RB, TP53 [23], PTEN [24] et al. All these targets play crucial role as an intrinsic factor of gastric carcinogenesis. In our previous studies, we had demonstrated expression of miRNA 106b, miRNA 93 and miRNA 25 were significantly higher in gastric cancer cell lines [26], tumor tissues and plasma form gastric cancer patients [27]. In this study, we analyzed the significance of miRNA106b~25 on clinicopathological features and prognosis of gastric cancer patients via multivariate analysis.

Multivariate analysis is able to perform trade studies across multiple dimensions whereas taking into account the effects of all variables on the responses of interest, as well as clinical and pathological features and miRNAs expression in this study, and in purpose of extract the important variable for the classification. PCA is a technique of data dimensionality reduction [29]. It contains a series of mathematical procedure which transforms a range of correlated variables into a (smaller) number of uncorrelated variables defined PCs, thus PCs is integrated to extract the main information of data sets. Scattered plots of PCA can show the biological alterations behind the data sets appropriately. The high coincidence of PCs and similarities in PC element could be achieved indifferent samples within similar pathological or pathophysiological status, so as to, these coincident constituents is in similar location in PCA scattered plots. In this study, scattered plots showed that the plasma and tumor miRNA 106b~25 expression, T stage, N stage, TNM stage were in similar location and correlated with each other.

PCA is a foundation for the algorithm of PLS-DA. PLS-DA is a partial least squares regression of binary variables, which can analyze the categories of a categorical variable (X) for predictor variables (Y). It is an coordination among the usual discriminant analysis and a discriminant analysis based on the significant PCs for the Y variables [12]. We used VIP and loading plots of PLS-DA analysis to identify the important X variables for the miRNA 106b~25s, it was showed that T stage, N stage, TNM stage were important factors to plasma and tumor miRNA 106b~25 expression.

In prognostic analyze, we used Kaplan–Meier analysis to prognostic univariate analysis. The Cox regression (or proportional hazards regression) is a statistical approach for investigating the effect of several variables upon the time a specified event takes to happen. In the context of an outcome such as death or disease progression this is known as Cox regression for survival analysis. The unique effect of a unit increase in a covariate is multiplicative with respect to the hazard rate. In this study, the cox regression showed that T stage, plasma miRNA 106b and tumor miRNA 93 significant risk factors for overall survival, the HR was 0.400, 0.371,0.295, respectively.

## Conclusion

miRNA 106b~25 expression in plasma and tumor correlated with T stage, N stage, TNM stage. miRNA 106b~25s expression increasing was important characters carried in the progressing stage of gastric cancer. T stage, plasma miRNA 106b and tumor miRNA 93 significant risk factors for overall survival.

## Additional file

**Additional file 1: Table S1.** The expression of miRNA 106b~25 in TCGA database.

## Data Availability

The datasets used during the current study are available from the corresponding author on reasonable request.
